# Wearable Monitoring Captures Sleep Disturbances in Patients With Chronic Inflammatory Demyelinating Polyneuropathy

**DOI:** 10.1111/jns.70069

**Published:** 2025-10-20

**Authors:** Jan Voth, Charlotte von Gall, Noëmi Gmahl, Noah M. Werner, Gerd Meyer zu Hörste, Sven G. Meuth, Marc Pawlitzki, Lars Masanneck

**Affiliations:** ^1^ Department of Neurology Medical Faculty and University Hospital Düsseldorf, Heinrich Heine University Düsseldorf Germany; ^2^ Institute of Anatomy II, Faculty of Medicine University Hospital, Heinrich Heine University Düsseldorf Germany; ^3^ Department of Neurology University Hospital Münster Münster Germany; ^4^ Hasso Plattner Institute, Digital Engineering Faculty University of Potsdam Potsdam Germany

**Keywords:** CIDP, sleep efficiency, sleep monitoring, WASO, wearables

## Abstract

**Background and Aims:**

Previous studies suggest that patients with chronic inflammatory demyelinating polyneuropathy (CIDP) experience impaired sleep, contributing to fatigue. Traditional methods like polysomnography or questionnaires are resource‐intensive and may not capture sleep in natural settings. We explored whether widely available consumer‐grade smartwatches offer a feasible way to assess sleep quality in this population.

**Methods:**

The Electronic Monitoring of Disease Activity in patients with CIDP (EMDA‐CIDP) study was a prospective observational study conducted from January 2023 to July 2024 at the University Hospitals of Düsseldorf and Münster. 46 patients had nighttime sleep recorded for 6 months via smartwatch. Additionally, clinical scores (e.g., Inflammatory Rasch‐built Overall Disability Scale), sleep (PSQI, Pittsburgh Sleep Quality Index), and quality of life (QoL) questionnaires were collected every 3 months.

**Results:**

Of 46 participants, 40 met adherence criteria (≥ 75% wear time on ≥ 75% of nights, median age: 66 years [IQR: 59.5–70.3], 9 [22.5%] female). Median PSQI score was 6 (4–7.6), sleep efficiency 93% (92–95), and WASO (wake after sleep onset) 32 min (24–42). Smartwatch‐derived objective sleep measures – sleep efficiency and WASO – correlated significantly with PSQI (Spearman's *R* = −0.49, *R* = 0.40), clinical scores, and QoL.

**Interpretation:**

Sleep is impaired in patients with CIDP and contributes to the overall disease burden. Our findings suggest that sleep disturbances can be tracked longitudinally using smartwatch‐derived markers. Integrating digital health data presents promising opportunities for long‐term sleep monitoring in this population. Larger studies, ideally incorporating polysomnography, are warranted to validate these findings.

## 1 Introduction

Chronic inflammatory demyelinating polyneuropathy (CIDP) is a progressive or relapsing disorder developing a heterogeneous disease course with symptoms persisting longer than 8 weeks [1]. Patients typically present with classic symptoms such as paresthesia, dysesthesia, symmetrical muscle weakness, neuropathic pain, gait disturbances, and hypo‐ or areflexia [2]. In addition, they often suffer from fatigue and count the latter among their most limiting symptoms [3]. Fatigue occurs not only in CIDP patients with active disease status, but also affects many patients in remission, and therefore represents a long‐term problem [4]. Furthermore, it is associated with patients' sleep quality and clinical disability [4]. In line with this, patients with CIDP also appear to exhibit generally increased sleep impairments resulting in reduced sleep quality [5].

Sleep quality is a multidimensional concept influenced by various physiological and behavioral factors [6, 7]. Since adequate sleep is important for regeneration, energy balance, cognition, mood, and overall quality of life (QoL) [6, 7, 8], poor sleep can impair both physical and mental health. Patients with neurological diseases such as CIDP appear to be particularly susceptible to poor sleep [4, 9], suggesting that improving sleep may help reduce or prevent symptoms like fatigue and depression. Many factors contribute to restful sleep, such as duration, efficiency and structural organization of sleep stages [7]. Key measures in sleep studies are sleep efficiency, defined as total sleep time divided by total time in bed, and wake after sleep onset (WASO) [6]. However, objective measurement of sleep quality is challenging and is usually performed using polysomnography in sleep laboratories, marked by an artificial environment and high resource requirements [10]. Therefore, in clinical studies, subjective sleep quality is usually assessed using standardized questionnaires, whose evaluation is time‐consuming yet may be essential for developing appropriate interventions [10, 11].

With the advent of various technologies – including wearables, nearables, and airables – it is now possible to monitor sleep outside of the sleep laboratory. Digital devices are becoming increasingly prevalent in society and are also gaining prominence in medical research [12, 13]. Many individuals already own such devices, particularly in the form of wearables [14]. While these devices have limitations in accurately capturing sleep – tending to overestimate total sleep time – they have demonstrated a relatively good ability to approximate sleep stages [15]. They therefore offer a solid and resource‐efficient way to collect objective sleep data under real‐life conditions [16]. Although a wide range of wearable devices is available on the market, relatively few studies have rigorously validated their performance, especially in orphan diseases like CIDP [17].

Given its high prevalence and relevance in this condition, sleep disturbances and sleep quality warrant further investigation in CIDP patients, especially regarding the multidimensional impact of sleep. With wearable technologies becoming increasingly accessible, their potential to capture meaningful sleep metrics in this population should be explored. As part of the electronic monitoring of disease activity in patients with chronic inflammatory demyelinating polyneuropathy (EMDA CIDP) prospective cohort study [18, 19], we assessed sleep via self‐reported established scales and widely available consumer‐grade wearable smartwatches and correlated both to disease severity and QoL. We hypothesize that objective sleep parameters, as measured by wearables, can predict subjective sleep quality and are associated with clinical status.

## 2 Methods

### 2.1 Study Design

The EMDA CIDP trial (NCT05723848) was conducted at the university hospitals of Düsseldorf and Münster in Germany, recruiting participants from January 2023 to April 2024. The detailed study design has already been explained in previous papers of the EMDA study and is outlined here in excerpts [18, 19]. For inclusion, patients had to meet the 2021 EAN/PNS CIDP criteria [2] (modified from the 2010 criteria in the original protocol), have documented evidence of a positive response to intravenous immunoglobulin (IVIG) therapy, and had to be under stable IVIG treatment for at least 8 weeks. CIDP variants were excluded. All eligible patients who opted to participate were enrolled in the study.

After giving written consent, patients were provided with a smartwatch (Scanwatch, Withings, Issy‐les‐Moulineaux, France) and set up with their smartphone. They were then instructed to wear the smartwatch day and night to record the longitudinal sleep data in their normal daily routine. Variables extracted from the sleep data include time to sleep, total time in bed, total time asleep, duration of different sleep stages, number of times woken up, WASO, sleep efficiency, and the Withings sleep score. Good sleep quality is marked by a sleep efficiency of at least 0.85 and a WASO of no more than 20 min [6]. Furthermore, the smartwatch was used to record longitudinal activity data [19].

**FIGURE 1:**
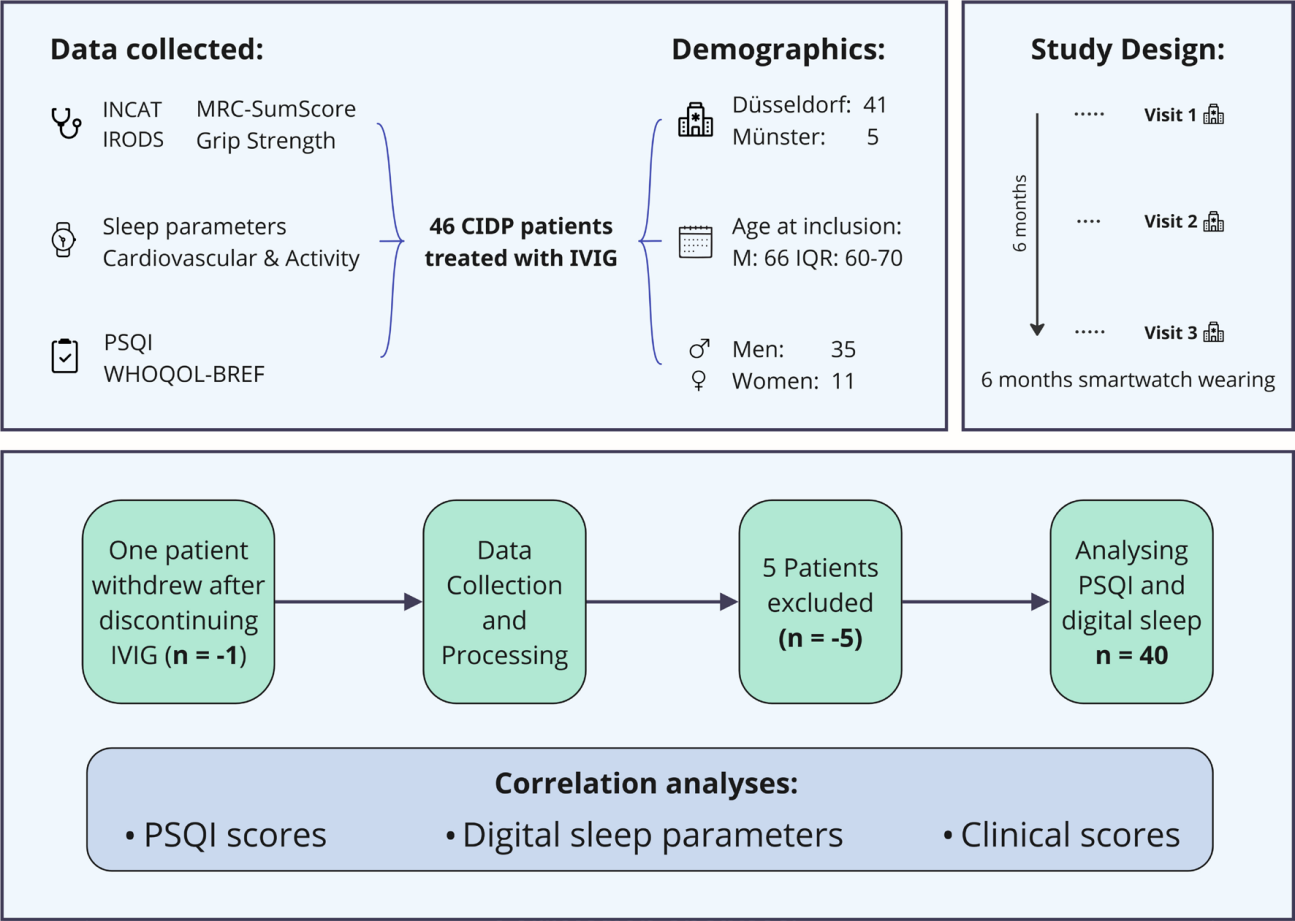
Overview of study and analysis. This figure illustrates the study design, key demographics, and the analysis workflow of the sleep assessment in the EMDA CIDP study. At the university hospitals of Düsseldorf and Münster, clinical scores, digital smartwatch data, and questionnaires were collected from 46 CIDP patients undergoing IVIG therapy. One patient discontinued due to stopping IVIG, and five were excluded due to insufficient nightly smartwatch adherence, leaving 40 patients for analysis. The analyses focused on the relationship between digital sleep data, PSQI scores, and clinical assessments. CIDP, chronic inflammatory demyelinating polyneuropathy; INCAT, Inflammatory Neuropathy Cause and Treatment disability scale; IQR, interquartile range; I‐RODS, Inflammatory Rasch‐built Overall Disability Scale; IVIG, intravenous immunoglobulin; M, median; MRC, Medical Research Council; *n*, number; PSQI, Pittsburgh Sleep Quality Index; WHOQOL‐BREF, World Health Organization Quality of Life – abbreviated version.

The clinical scores and questionnaires outlined below were recorded during study visits at baseline (V1), after 3 months (V2), and at the end of the study (after 6 months, V3). Variations in these time points resulted from differing IVIG intervals and scheduling constraints in clinical practice. See Figure 1 for the study overview.

### 2.2 Clinical Scores and Questionnaires

The Medical Research Council (MRC) sum score [20] was used to assess muscle strength across six muscle groups with a maximum score of 60. Grip strength [21, 22] was measured using a Martin Vigorimeter, ranging from 0 to 160 kPa. The Inflammatory Rasch‐built Overall Disability Scale (I‐RODS) [23, 24] measures 24 daily activities. Each item is rated from 0 (impossible) to 2 (easily performed), resulting in a total score between 0 and 48. The Inflammatory Neuropathy Cause and Treatment (INCAT) disability scale [25] evaluates arm and leg disability on a 10‐point scale. A score of 0 indicates full function.

The Pittsburgh Sleep Quality Index (PSQI) [26] questionnaire was used to assess subjective sleep quality, generating a global score from 0 to 21, with higher scores indicating poorer sleep. A score > 5 is considered poor sleep [26]. The global score comprises seven components: subjective sleep quality (C1), sleep latency (C2), sleep duration (C3), habitual sleep efficiency (C4), sleep disturbances (C5), use of sleep medication (C6), and daytime dysfunction (C7), each ranging from 0 to 3. The items 5a (contributing to C2) and 5b–5j (contributing to C5) cover the following aspects: difficulty falling asleep (5a), waking up at night or early morning (5b), having to use the bathroom (5c), trouble breathing comfortably (5d), coughing or snoring (5e), feeling too cold (5f), feeling too hot (5 g), having bad dreams (5 h), having pain (5i), and other reasons (5j). The Abbreviated World Health Organization Quality of Life (WHOQOL‐BREF) [27, 28] assessment evaluates QoL in four key domains: social relationships, environment, physical health, and psychological health. It offers fast assessment for clinical use and research across different health conditions [27, 29]. In the subsequent analyses, the median of clinical outcomes across all three visits was used.

### 2.3 Statistical Analysis

Analyses and visualization were conducted using Graph Pad Prism (Version 10.4.1, GraphPad Software, Boston, MA, USA) as well as Python 3.9.11 (Python Software Foundation, Delaware, USA) working with the following packages: pandas 2.1.1, numpy 1.26.0, matplotlib 3.8.0, seaborn 0.13.2, scipy 1.14.0, statsmodels 0.14.4, and openpyxl 3.1. A web application for synchronizing, organizing, and visualizing data was developed using the Dash framework version 2.6.1.

### 2.4 Adherence Calculation and Criteria

After reviewing and analyzing the average reported sleep habits, a night cycle was defined as the period from 10:00 P.M. to 8:00 A.M. Adherence was then defined as wearing the smartwatch for at least 75% of the night cycle duration. Patients were excluded from the analysis if their smartwatch adherence was not met on less than 75% of the days throughout the study period.

### 2.5 Distribution, Correlation and Regression Analyses

Distribution of normality was assessed using the Shapiro–Wilk and D'Agostino–Pearson tests. Data were considered normally distributed if both *p*‐values were above 0.05, in which case the mean and standard deviation (SD) were reported. If any *p*‐value was ≤ 0.05, the data were considered nonnormally distributed, and the median was reported with the interquartile range (IQR) instead.

For correlation analyses, Spearman's correlation (coefficient represented as R throughout the manuscript) was utilized since at least one of the analyzed variables was not normally distributed and metric. Spearman's coefficients were interpreted according to Chan [30]. Multiple testing was corrected according to the Benjamini‐Hochberg procedure. To visualize trends in the relationship between PSQI and selected digital and clinical parameters, linear regression was used to generate trendlines, even if certain assumptions were not fully met. Group comparisons of continuous, nonnormally distributed variables were performed using the nonparametric Mann–Whitney *U* test [31], with effect sizes reported as *r* and significance set at *p* < 0.05 [32].

### 2.6 Ethical Considerations

Ethical approval was granted by the Ethics Committees of Düsseldorf (No.: 2022‐1881_1) and Münster (No.: 2023‐106‐b‐S). All participants gave informed consent, and all study procedures adhered to the principles of the Declaration of Helsinki.

## 3 Results

For the EMDA‐CIDP trial, a total of 46 patients were recruited, of which 41 patients were enrolled in Düsseldorf and five patients in Münster. One patient withdrew from the study due to discontinuation of IVIG therapy, and an additional five patients were excluded from the analyses due to not meeting adherence criteria (Figure 1). This resulted in a total sample size of *n* = 40, including 9 (22.5%) females and 31 (77.5%) males. At study inclusion, the median age was 66 years (59.5–70.3), and the median disease duration was 4 years (1–9). For a detailed cohort description, including an overview of sleep parameters, see Table 1. The median smartwatch adherence during the night was 98.6% (95.8–99.7). Further details on adherence to each user can be seen in Figure S1.

**TABLE 1:** Trial cohort characteristics.

		Missing/*n*	Overall
*n* (%)		0/46	46 (100.0)
Study center, *n* (%)	MS	0/46	5 (10.9)
	DD		41 (89.1)
Patients study dropout, *n* (%)		0/46	6 (13.0)
Sex, *n* (%)	w	0/40	9 (22.5)
	m		31 (77.5)
Age at inclusion [y], median [Q1, Q3]		0/40	66 [59.5, 70.3]
Age at diagnosis [y], median [Q1, Q3]		0/40	59 [53.5, 65.5]
Disease duration at inclusion [y], median [Q1, Q3]		0/40	4 [1, 9]
Median INCAT Disability Score V1‐3, median [Q1, Q3]		0/40	2 [1, 4]
Median I‐RODS V1‐3, median [Q1, Q3]		0/40	41 [30.8, 44]
Median MRC sum score V1‐3, median [Q1, Q3]		0/40	55 [50, 59]
Median PSQI results, median [Q1, Q3]		0/40	6 [4, 7.6]
Duration of immunoglobulin therapy at inclusion [mo], median [Q1, Q3]		0/40	29.5 [8.5, 86.8]
Immunoglobulin treatment interval [d], median [Q1, Q3]		0/40	28 [28, 42]
BMI (kg/m^2^), mean [SD]		0/40	28.1 [3.9]
Duration in study [d], median [Q1, Q3]		0/40	175 [168, 203]
% of hours with data from digital device per subject, median [Q1, Q3]		0/40	98.3 [96.9, 99.1]
Daily smartwatch adherence target for sleep analysis met, *n* (%)		0/46	40 (87.0)
Smartwatch sleep efficiency, median [Q1, Q3]		0/40	0.93 [0.92, 0.95]
Smartwatch WASO (min), median [Q1, Q3]		0/40	23 [14.5, 32]
Smartwatch total sleep time (min), median [Q1, Q3]		0/40	513 [487, 549]
Smartwatch sleep latency (min), median [Q1, Q3]		0/40	2 [2, 2]
Self‐reported sleep latency (min), median [Q1, Q3]		0/40	15 [10, 30]

Abbreviations: BMI, body mass index; d, day; DD, University Hospital Düsseldorf; I‐RODS, Inflammatory Rasch‐built Overall Disability Scale; INCAT, Inflammatory Neuropathy Cause and Treatment; m, men; min, minutes; mo, month; MRC, Medical Research Council; MS, University Hospital Münster; *n*, quantity; PSQI, Pittsburgh Sleep Quality Index; Q1, lower quartile; Q3, upper quartile; SD, standard deviation; V1–3, visit 1, 2, 3; w, women; WASO, wake after sleep onset; y, year.

### 3.1 Self‐Reported Sleep Quality

**FIGURE 2:**
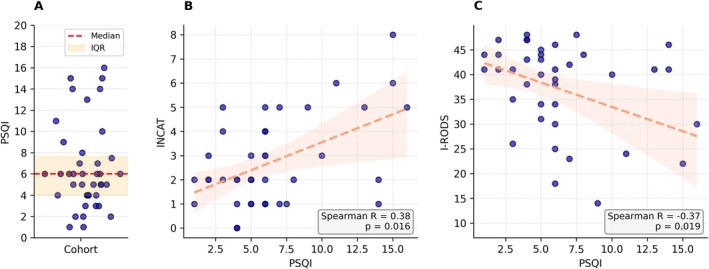
Self‐reported sleep quality and its association with clinical disability scores in CIDP patients. (A) Scatter plot of individual PSQI median scores across the cohort (*n* = 40), with median (red dashed line) and interquartile range (orange shading). Median was 6 (IQR: 4–7.6). (B) Scatterplot with regression line showing the relationship between PSQI median scores and median INCAT disability scores, including Spearman correlation coefficient and *p*‐value. (C) Scatterplot with regression line showing the relationship between PSQI median scores and median I‐RODS scores, including Spearman correlation coefficient and *p*‐value. Scatter points are jittered for better visualization. Regression lines and 95% confidence intervals are shown in salmon color. Regression lines were only used to visualize correlations, since neither the PSQI nor the INCAT or I‐RODS values were normally distributed. CIDP, chronic inflammatory demyelinating polyneuropathy; INCAT, Inflammatory Neuropathy Cause and Treatment disability scale; I‐RODS, Inflammatory Rasch‐built Overall Disability Scale; PSQI, Pittsburgh Sleep Quality Index; IQR, interquartile range, *n*, number.

Self‐reported sleep quality showed a median PSQI score of 6 (4–7.6). A high proportion of the patients showed a PSQI score > 5, thus indicating poor sleep quality (Figure 2A). All components except component 6 (use of sleep medication) appeared to contribute equally to the global score (Figure S2A). Among the sleep disturbances, problems falling asleep (5a), waking up at night or early morning (5b), having to use the bathroom (5c), and pain (5i) most frequently showed higher scores (Figure S2B).

### 3.2 Objective Measures for Sleep Quality

Digital sleep data captured via smartwatches showed the following results: Patients slept a median of 513 min (487–549) per night, spent a median of 554 min (519–587) in bed, and spent a median of 263 min (233–294) in light and 243 min (205–272) in deep sleep. They showed a median sleep efficiency of 93% (92–95) and a median WASO of 23 min (14.5–32) (Figure S3).

### 3.3 Correlations Between Subjective Sleep Quality and Disability Scores

To explore relationships within the large dataset, we first conducted multiple correlation analyses. Notably, the disability measurement scales I‐RODS and INCAT as clinical parameters showed meaningful associations with subjective sleep quality and digital sleep markers. MRC sum score and grip strength showed less relatedness. Therefore, we correlated PSQI with INCAT and I‐RODS as shown in Figure 2B,C. The Spearman correlation showed a fair positive and a fair inverse correlation between clinical scores and PSQI (INCAT: *R* = 0.38, *p* = 0.016; I‐RODS: *R* = −0.37, *p* = 0.019), indicating poorer subjective sleep at higher disease severity. Stratifying the cohort by disability scores revealed significantly poorer PSQI outcomes in the more affected patients. Those with I‐RODS < 35 (*n* = 13) had a median PSQI of 6 (6–11) versus 5 (3.5–6.5) in patients with I‐RODS ≥ 35 (Mann–Whitney *U* = 96.5, *p* = 0.022, *r* = 0.36). Similar significant differences were observed when using INCAT scores, with patients scoring > 3 (*n* = 12) showing higher PSQI values than those scoring ≤ 3 (median PSQI: 8 [6–13.5] vs. 5 [4–6]; Mann–Whitney *U* = 83.0, *p* = 0.012, *r* = 0.4; Figure S4).

### 3.4 Correlations Between Digital Sleep Markers With Subjective Sleep Quality and Clinical Scores

**FIGURE 3:**
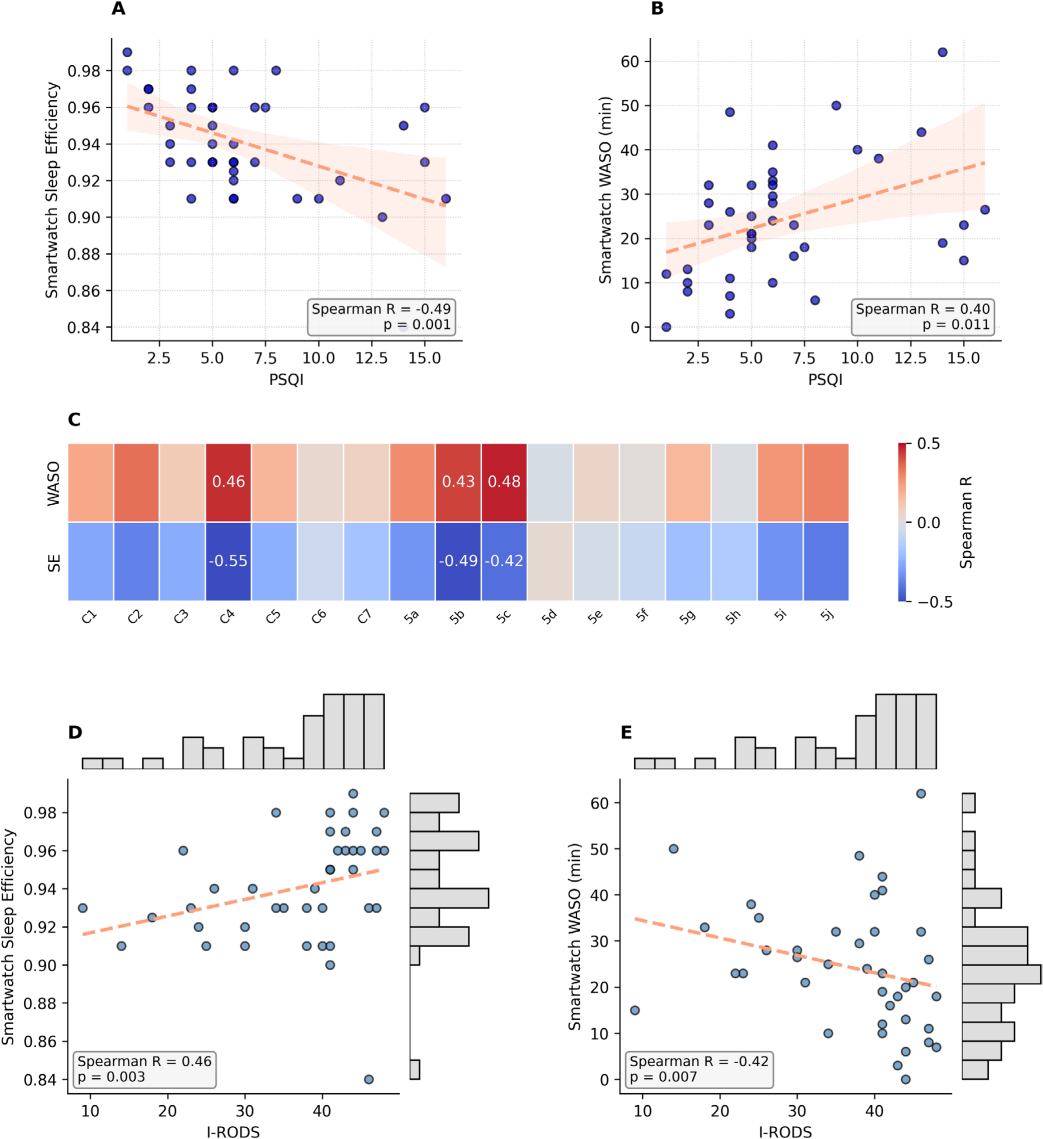
Associations between digital sleep parameters, PSQI, and clinical scores in CIDP patients. (A, B) Scatterplots with regression lines showing relationships between PSQI median scores and median smartwatch‐derived sleep efficiency (A) and WASO (B), respectively, with Spearman correlation coefficients and *p*‐values. Regression lines were only used to visualize correlations, since neither the PSQI nor the sleep efficiency or WASO values were normally distributed. (C) Heatmap of Spearman correlations between digital sleep metrics (median SE, median WASO) and PSQI subcomponents (median scores C1–C7 and 5a–5j) after false discovery rate correction using the Benjamini‐Hochberg method; significant correlations are annotated. (D, E) Jointplots illustrating associations between median I‐RODS scores and digital sleep efficiency (D) or WASO (E), including regression lines, marginal histograms, and Spearman statistics. Regression lines were only used to visualize correlations, since neither the I‐RODS nor the sleep efficiency or WASO values were normally distributed. Scatter points are jittered for better visualization. Regression lines and 95% confidence intervals are shown in salmon color. CIDP, chronic inflammatory demyelinating polyneuropathy; I‐RODS, Inflammatory Rasch‐built Overall Disability Scale; PSQI subcomponents: C1, subjective sleep quality; C2, sleep latency (including 5a: falling asleep); C3, sleep duration; C4, sleep efficiency; C5, sleep disturbance; C6, use of sleep medication (*n* = 5); C7, daytime dysfunction; C5 subcomponents: 5b, wake up in the middle of the night or early morning; 5c, have to get up to use the bathroom; 5d, cannot breathe comfortably; 5e, cough or snore loudly; 5f, feel too cold; 5g, feel too hot; 5h, have bad dreams; 5i, have pain; 5j, other reason(s); PSQI, Pittsburgh Sleep Quality Index; SE, sleep efficiency; WASO, wake after sleep onset.

We then moved to compare digital with self‐reported sleep markers and clinical measures (Figure S5). Among the digital markers, sleep efficiency as well as WASO emerged as prominent in early analyses showing the strongest correlation with patients' PSQI scores (sleep efficiency: *R* = −0.49, *p* = 0.001; WASO: *R* = 0.40, *p* = 0.011). An increased PSQI and therefore poor subjective sleep quality corresponded to lower sleep efficiency and higher WASO (Figure 3A,B). Additionally, both parameters showed a relation with PSQI's subscores. They exhibited significant correlation with C4 (sleep efficiency), 5b (waking up at night or early morning), and 5c (having to use the bathroom). In addition, they displayed an association with C2 (sleep latency) and 5i (having pain) (Figure 3C).

Regarding clinical scores, higher I‐RODS values and thus lower disability were associated with higher sleep efficiency (*R* = 0.46, *p* = 0.003; Figure 3D), and lower WASO (*R* = −0.42, *p* = 0.007; Figure 3E). Similarly, higher INCAT scores and thus higher disability were associated with lower sleep efficiency (*R* = −0.39, *p* = 0.014), and higher WASO (*R* = 0.34, *p* = 0.033; Figure S6A,B).

### 3.5 Correlations Between Daily Activity and Sleep Quality

In the next step, we investigated the relation between patient activity, recorded via smartwatch, and their subjective and objective sleep quality. In the preliminary correlation analyses, we correlated the various activity markers with PSQI and digital sleep markers in a heat map, similar to Figure S5. Moderate activity emerged as the most suitable marker, prompting us to investigate its direct associations further. Moderate activity showed a moderate negative correlation with the PSQI (*R* = −0.36, *p* = 0.022) and appeared to be related primarily to its subcategory of C1, subjective sleep quality (*R* = −0.38, *p* = 0.015) and C2, sleep latency (*R* = −0.33, *p* = 0.038) (Figure S7). However, moderate activity did not show a significant correlation with either sleep efficiency or WASO.

### 3.6 Quality of Life and Quality of Sleep

**FIGURE 4:**
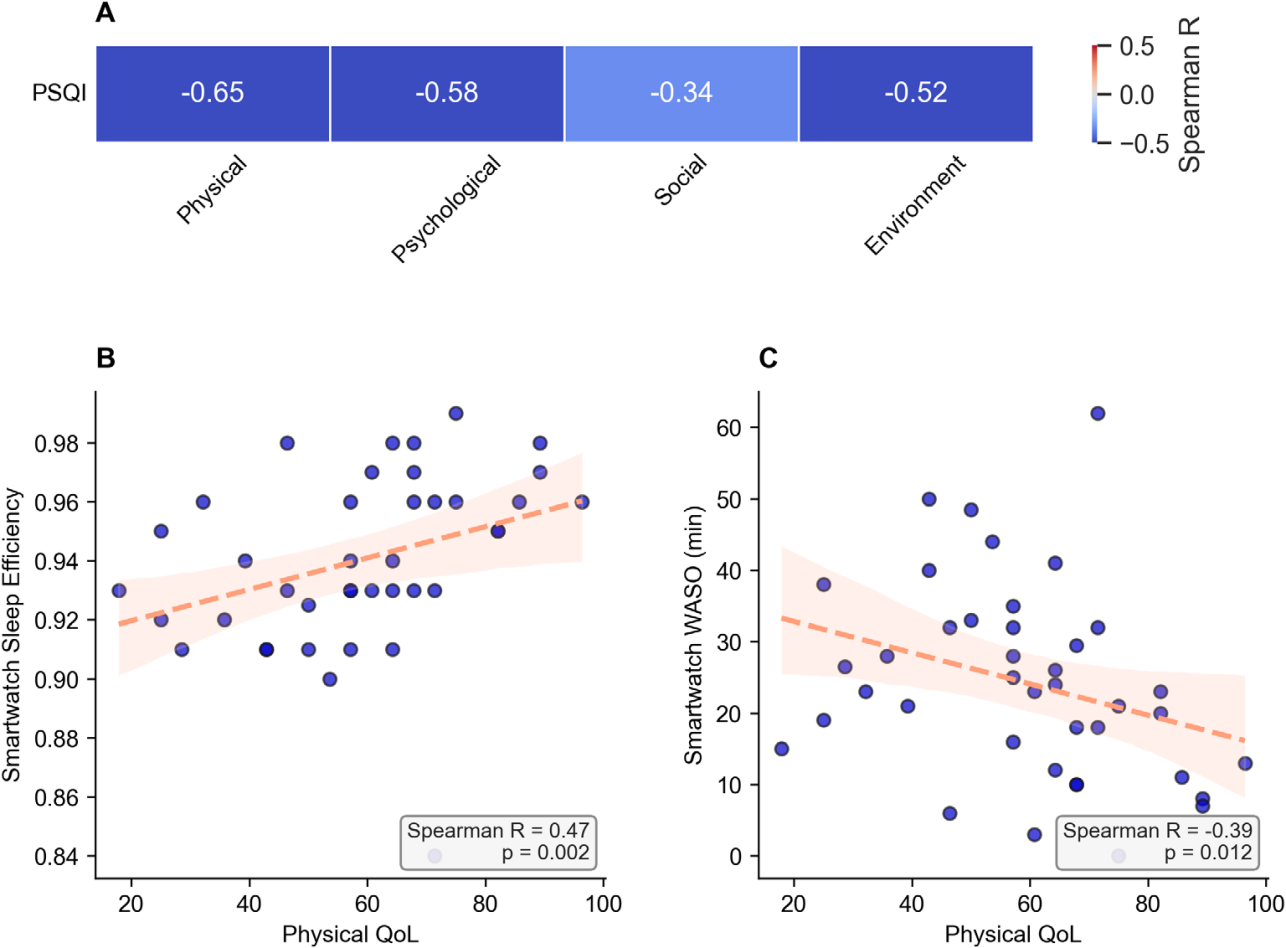
Associations between digital sleep parameters, PSQI, and quality of life domains in CIDP patients. (A) Heatmap of Spearman correlation coefficients between the PSQI median score and QoL domains (Physical Health, Psychological Health, Social Relationships, Environment). Correlations are adjusted for multiple testing using the Benjamini‐Hochberg false discovery rate correction. Significant correlations are annotated. QoL was assessed using WHOQOL‐BREF. (B, C) Scatterplots with regression lines illustrating relationships between median Physical Health QoL and median smartwatch‐derived sleep efficiency (B) and WASO (C), respectively, with Spearman correlation coefficients and *p*‐values displayed. Regression lines and 95% confidence intervals are shown in salmon color. Regression lines were only used to visualize correlations, since neither the I‐RODS nor the WASO values were normally distributed. CIDP, chronic inflammatory demyelinating polyneuropathy; PSQI, Pittsburgh Sleep Quality Index; QoL, quality of life; WASO, wake after sleep onset; WHOQOL‐BREF, World Health Organization Quality of Life – abbreviated version.

Finally, we analyzed the QoL within the cohort. The median was 60.7 in the physical health domain, 75.0 in psychological health, 66.7 in social relationships, and 81.3 in the environmental domain (Figure S8). Notably, the domains of physical health and social relationships showed a discernible decline compared to a field study cohort from WHO with healthy subjects [27]. Higher scores in all four domains were significantly associated with lower PSQI global scores (Figure 4A), thus indicating better subjective sleep quality. Similarly, higher scores in all four domains were associated with higher sleep efficiency and lower WASO (Figure S9). In particular, higher physical health scores correlated significantly with higher sleep efficiency (*R* = 0.47, *p* = 0.002) and lower WASO (*R* = −0.39, *p* = 0.012), see Figure 4B,C.

## 4 Discussion

Sleep disturbances are highly relevant yet understudied in chronic inflammatory disorders of the peripheral nervous system. In this study, we used a multimodal approach to characterize sleep‐related impairments in patients with CIDP, demonstrating that sleep dysfunction represents a significant but often overlooked aspect of the disease burden. Moreover, our findings demonstrate that wearable‐based digital sleep can reflect patients' self‐reported sleep quality in patients with CIDP and correlate with clinical disease severity. High wearable adherence in this cohort demonstrates the feasibility of digital monitoring even among older patients and longer time periods, supporting its wider implementation in clinical practice.

As previous studies have already highlighted, a large proportion of patients with CIDP suffers from disturbances in nocturnal sleep [5, 33]. Although this issue has now been confirmed by different studies, it has yet to be reflected in current clinical guidelines and recommendations [2]. The prominent self‐reported issues identified in our cohort – such as difficulties initiating sleep, prolonged sleep latency, and nocturnal pain – could be tackled more effectively than is currently often the case. Potential improvements include improved sleep hygiene, a constant bedtime routine, or regular exercise [7]. Moreover, the sleep‐related disturbances are closely linked to daytime dysfunction, adding an additional burden to patients who already face significant everyday disability. In line with the moderate disease burden observed under ongoing therapy, our study cohort also showed moderately impaired PSQI scores. However, stratifications according to clinical disability scores (I‐RODS, INCAT) revealed significantly poorer self‐reported sleep quality in patients with higher symptom burden. Improving sleep at night could significantly relieve the daily burden of these patients and may help prevent further comorbidities [7]. Our findings further suggest that improving sleep – for instance, through neuropathic pain management or increased physical activity – may not only enhance sleep quality but also positively impact clinical symptoms and overall QoL [34, 35]. Ultimately, clinicians treating CIDP patients should pay closer attention to sleep disturbances, especially in those with more severe disability and symptoms such as fatigue or increased motor exhaustion. While self‐reported questionnaires like the PSQI may provide an initial assessment, digital tools are likely to take on this role more prominently in the future.

Focusing now on wearable‐generated sleep data, the analysis highlighted WASO and sleep efficiency as the most relevant digital sleep metrics. In our CIDP cohort, smartwatch‐derived WASO values suggested reduced objective sleep quality, a finding that was supported by the self‐reported PSQI. Notably, the PSQI's sleep efficiency component (C4) also showed partly substantial impairments. Interestingly, smartwatch‐derived sleep efficiency indicated predominantly good sleep. This may be explained by the short smartwatch sleep latency compared to self‐reported values (Table 1), together with lower PSQI scores contrasted to more disabled CIDP cohorts – suggesting longer sleep duration and therefore reduced impact of interruptions on sleep efficiency. Despite this discrepancy, both digital parameters – WASO and sleep efficiency – were significantly correlated with self‐reported sleep quality directly, while offering the additional advantage of objective, longitudinal monitoring. Therefore, they could potentially detect or map sleep disorders – probably not limited to neuroimmunological diseases – with WASO possibly serving as a more reliable marker. Additionally, both parameters demonstrated associations with patients' QoL, underscoring their potential relevance as indicators of patient well‐being. Given the major advantage of consumer wearable technology's widespread usage and growing adoption, this might indeed help to objectify sleeping complaints typical for CIDP. While these simple devices cannot replace polysomnography, they can continuously approximate basic sleep stats in the patient's usual sleep environment at lower cost. Compared to self‐reported snapshots from patient‐reported outcome measures (PROMs), which can be time‐consuming to complete and process, wearables provide passive, continuous, and objective recordings that are usually readily accessible. In clinical settings, especially in more affected cases, a brief review of recorded sleep data instead of an additional questionnaire could provide a quick insight into sleep quality, serve as a bridge to address previously unreported sleep problems, and could in principle do so longitudinally.

Our study suffers several limitations. Firstly, the digital thresholds for good sleep quality defined by the National Sleep Foundation [6] were based on studies likely involving little or no smartwatch‐derived data – reducing their suitability for our results. This may also explain discrepancies in sleep quality, as measured by sleep efficiency and WASO. Moreover, potential comorbidities, including sleep apnea or affective disorders, were not accounted for in our analysis. Sleep disturbances and irregularities observed in this study could therefore also be influenced by these underlying conditions [36, 37]. Furthermore, general limitations of wearable devices must be acknowledged, including the above‐mentioned reduced performance in sleep tracking and, in some cases, lack of significant agreement with polysomnography [17, 38]. While some devices show significant agreement with actigraphy, their performance tends to be poorer in individuals with sleep disorders than in healthy populations [39, 40]. Finally, our CIDP cohort was relatively small, demographically homogeneous, and did not include CIDP variants, highlighting the need for larger studies. Moreover, future analyses should include polysomnographic or actigraphy assessments to further validate and strengthen our findings.

In conclusion, our findings highlight sleep disturbances as a relevant and underrecognized comorbidity in CIDP. Despite their clinical impact, these symptoms remain insufficiently addressed in current guidelines and are often overlooked in routine care. The use of digital health data offers promising opportunities to monitor sleep patterns also in combination with motor functional measures over time [19] and may help capture treatment‐related changes more effectively in real‐world settings.

## Conflicts of Interest

J.V. reports no conflicts of interest. C.v.G. reports no conflicts of interest. N.G. reports no conflicts of interest. N.M.W. reports no conflicts of interest. G.M.z.H. reports no conflicts of interest related to this study. He was supported by grants from the Deutsche Forschungsgemeinschaft (DFG) (ME4050/12‐1, ME4050/13‐1) and from the Bundesministerium für Bildung und Forschung (BMBF) “Lipid Immune Neuropathy Consortium”. S.G.M. reports no conflicts of interest related to this study. He has received honoraria for lecturing and travel expenses for attending meetings from Almirall, Amicus Therapeutics Germany, ArgenX, Bayer Health Care, Biogen, Celgene, Diamed, Genzyme, MedDay Pharmaceuticals, Merck Serono, Novartis, Neuraxpharm, Novo Nordisk, ONO Pharma, Roche, Sanofi‐Aventis, Chugai Pharma, QuintilesIMS, and Teva. His research is funded by the German Ministry for Education and Research (BMBF), Bundesinstitut für Risikobewertung (BfR), Deutsche Forschungsgemeinschaft (DFG), Else Kröner Fresenius Foundation, Gemeinsamer Bundesausschuss (G‐BA), German Academic Exchange Service, Hertie Foundation, Interdisciplinary Center for Clinical Studies (IZKF) Muenster, German Foundation Neurology, and by Alexion, Almirall, Amicus Therapeutics Germany, Biogen, Diamed, Fresenius Medical Care, Genzyme, HERZ Burgdorf, Merck Serono, Novartis, ONO Pharma, Roche, and Teva, all outside the scope of this study. M.P. reports no conflicts of interest related to this study. He has received honoraria for lecturing and travel expenses for attending meetings from Alexion, ArgenX, Bayer Health Care, Biogen, Demecan, Hexal, Merck Serono, Neuraxpharm, Novartis, Roche, Sanofi‐Aventis, Takeda, and Teva. His research is funded by ArgenX, Biogen, Hexal, and Novartis, B. Braun Stiftung and by the German Multiple Sclerosis Foundation (DMSG). L.M. reports no conflicts of interest related to this study. He has received honoraria for lecturing, consulting, and travel expenses for attending meetings from Biogen, Merck, Sanofi, ArgenX, Roche, Alexion, and Novartis, all outside the scope of this work. His research is funded by the German Multiple Sclerosis Foundation (DMSG) and the Deutsche Forschungsgemeinschaft (DFG, German Research Foundation)—493659010.

## Supporting information


**Data S1:** jns70069‐sup‐0001‐Figures.pdf.

## Data Availability

The datasets generated and analyzed during the current study are available from the corresponding author upon reasonable request. All inquiries will be addressed within 6 weeks and must comply with the data protection regulations of the University Hospitals Düsseldorf and Münster.
