# Supplementary material for: Wearable Monitoring Captures Sleep Disturbances in Patients With Chronic Inflammatory Demyelinating Polyneuropathy
Source: J Peripher Nerv Syst. 2025 Oct 20;30(4):e70069. doi: 10.1111/jns.70069 (PMC12536332; doi:10.1111/jns.70069)
Supplement: Supplementary file 1 — Data S1: jns70069‐sup‐0001‐Figures.pdf. [file JNS-30-0-s001.pdf]

Supplement

Supplemental Figure 1

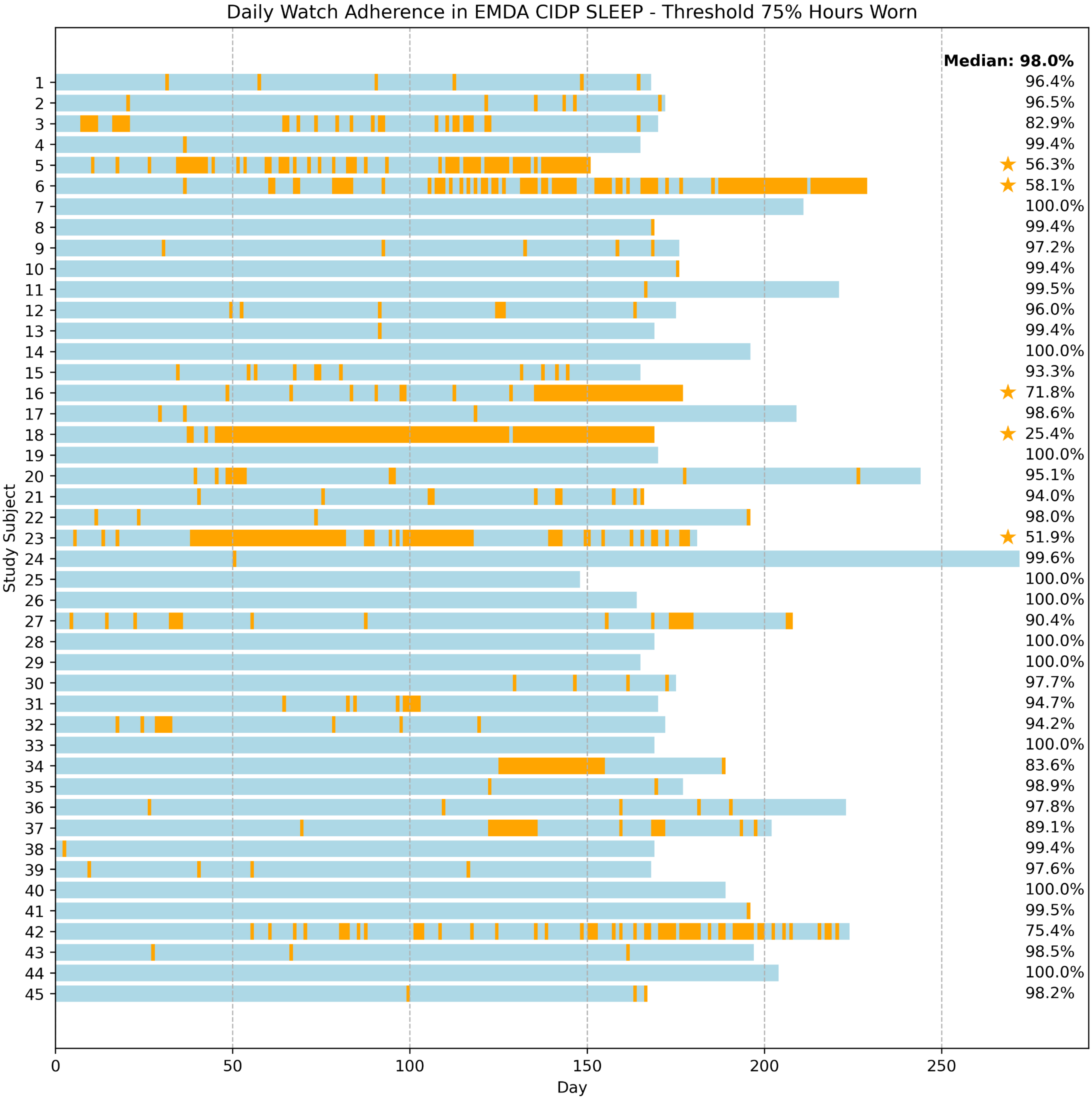

Suppl. Figure 1: Nightly smartwatch adherence in the study cohort.

This bar plot illustrates nightly adherence to smartwatch wearing across the entire study period for each patient. Nighttime was defined as the interval between 10:00 PM and 8:00 AM. A night was classified as "adherent" if the smartwatch was worn for at least 75% of that time window. Adherent nights are shown in light blue, and non-adherent nights are shown in orange. Patients marked with an orange star did not meet the overall adherence threshold of 75% of nights and were excluded from the analyses. The percentage of adherent nights is displayed on the right for each patient. The median adherence across all patients was 98%.

EMDA CIDP: Electronic monitoring of disease activity in patients with chronic inflammatory demyelinating polyneuropathy,

Supplemental Figure 2

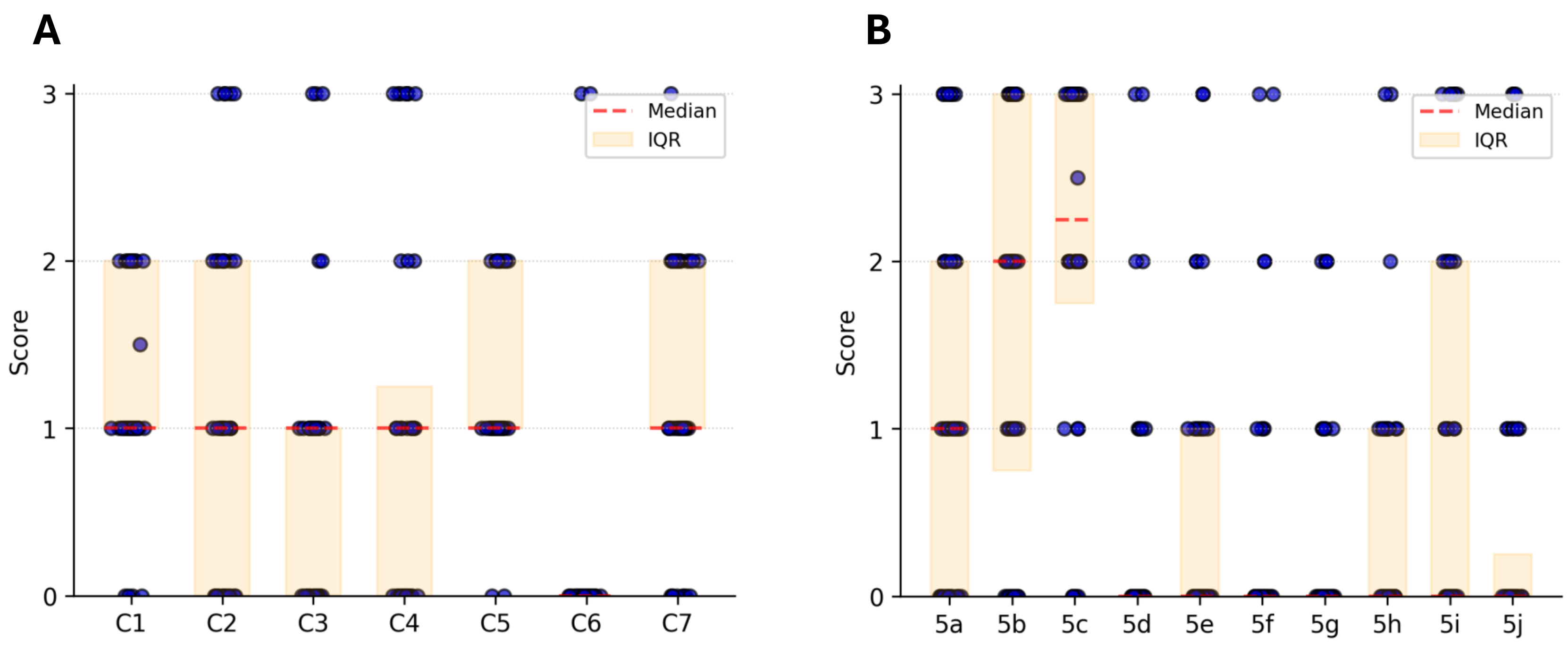

Suppl. Figure 2: Distributions of PSQI subcomponent scores (C1–C7 and C5 subcomponents) in CIDP patients.

(A) Dot plots showing the distributions of median scores for the seven PSQI subcomponents (C1–C7).

(B) Dot plots for the median individual items contributing to PSQI subcomponent 5a-5j (contribution to component C5, sleep disturbances)

Each dot represents one patient. Red dashed lines indicate the group median; shaded orange areas represent the interquartile range. Horizontal jitter was applied to improve visualization.

CIDP: Chronic inflammatory demyelinating polyneuropathy, PSQI: Pittsburgh Sleep Quality Index, C1: Subjective sleep quality, C2: Sleep latency (including 5a: Falling asleep), C3: Sleep duration, C4: Sleep efficiency, C5: Sleep disturbance, C6: Use of sleep medication (n=5), C7: Daytime dysfunction; C5 subcomponents: 5b: Wake up in the middle of the night or early morning, 5c: Have to get up to use the bathroom, 5d: Cannot breathe comfortably, 5e: Cough or snore loudly, 5f: Feel too cold, 5g: Feel too hot, 5h: Have bad dreams, 5i: Have pain, 5j: Other reason(s)

Supplemental Figure 3

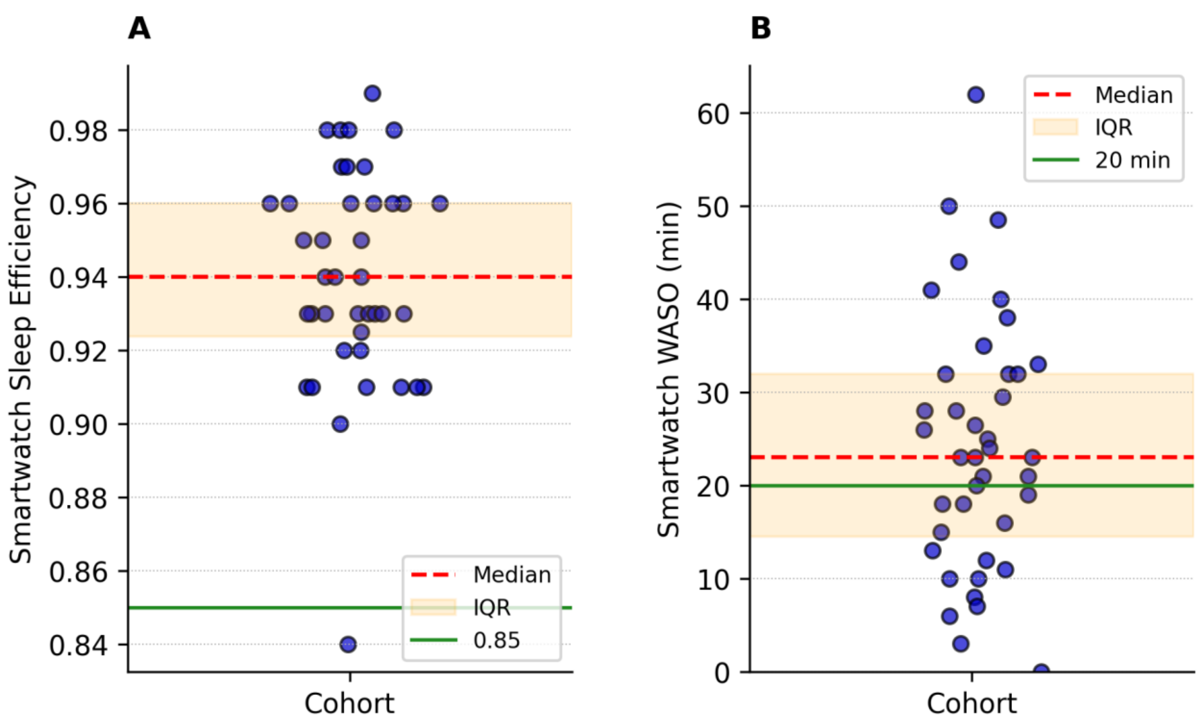

Suppl. Figure 3: Distributions of smartwatch-derived sleep efficiency and WASO in CIDP patients.

Dot plots showing the distributions of smartwatch-derived sleep efficiency (**A**) and WASO (**B**) in the CIDP cohort. Each dot represents one patient. Red dashed lines indicate the group median; shaded orange areas represent the interquartile range (IQR). Horizontal threshold lines are shown at sleep efficiency = 0.85 and WASO = 20 min, based on National Sleep Foundation's criteria indicating good sleep quality at sleep efficiency ≥ 0.85 and WASO ≤ 20 min. Horizontal jitter was applied to enhance visualization.

CIDP: Chronic inflammatory demyelinating polyneuropathy, WASO: Wake After Sleep Onset.

Supplemental Figure 4

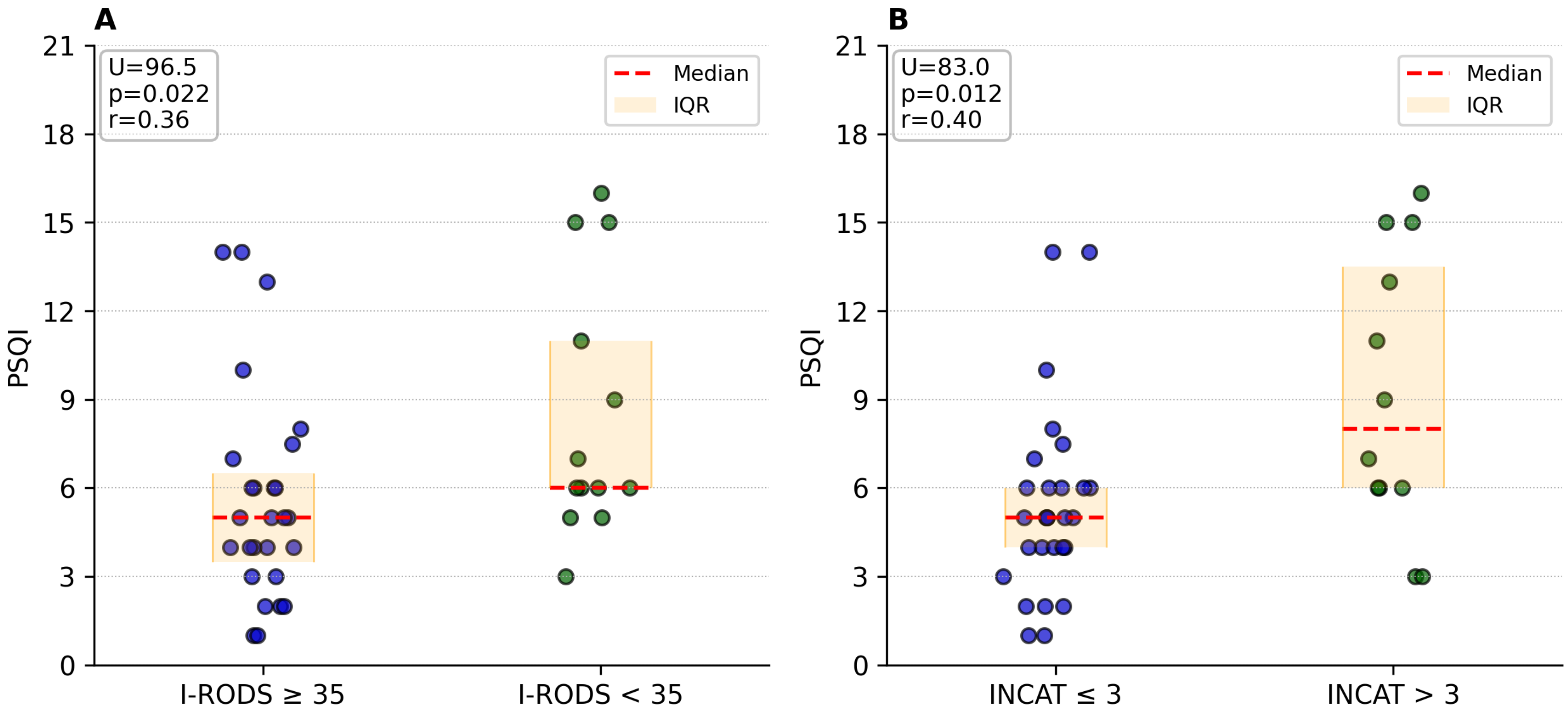

Suppl. Figure 4: Distributions of PSQI scores stratified by I-RODS and INCAT in CIDP patients.

Dot plots showing the distributions of PSQI scores in the CIDP cohort, divided according to I-RODS (A) and INCAT (B) groups. Each dot represents one patient. Red dashed lines indicate the group median; shaded orange areas represent the interquartile range (IQR). Mann-Whitney U-test statistics (U, p-value, and effect size r) are shown in the upper-left corner of each plot. Horizontal jitter was applied to enhance visualization.

CIDP: Chronic inflammatory demyelinating polyneuropathy, PSQI: Pittsburgh Sleep Quality Index, IQR: Interquartile range, I-RODS: Inflammatory Rasch-built Overall Disability Scale, INCAT: Inflammatory Neuropathy Cause and Treatment disability scale.

Supplemental Figure 5

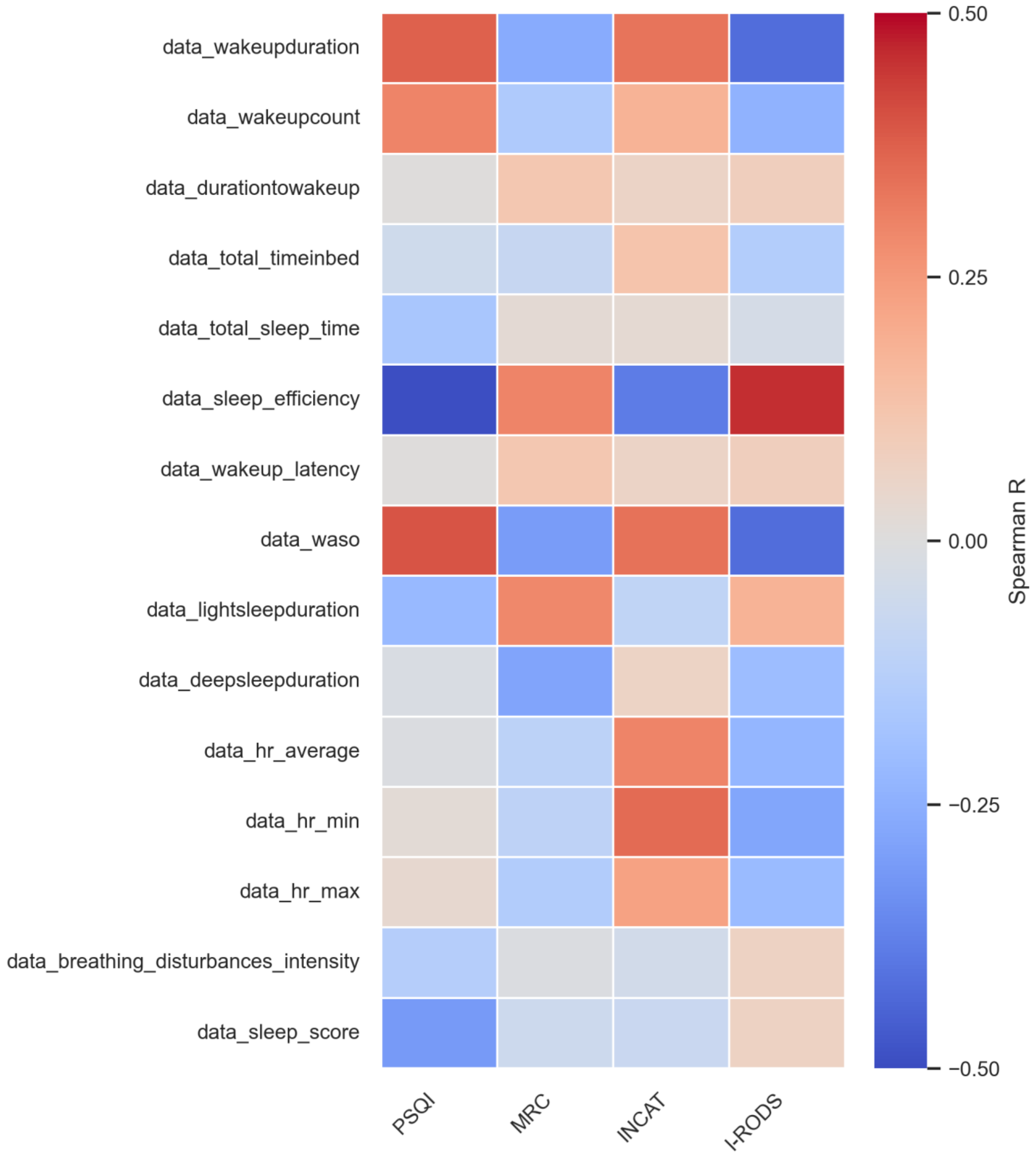

Suppl. Figure 5: Spearman correlations between digital sleep metrics, PSQI and clinical scores in CIDP patients after Benjamini-Hochberg correction for multiple testing.

Heatmap showing the correlation coefficients between various median digital sleep parameters derived from smartwatches and four clinical metrics: PSQI, MRC sum score, INCAT, and I-RODS. Color intensity reflects the strength and direction of correlation.

CIDP: Chronic inflammatory demyelinating polyneuropathy, PSQI: Pittsburgh Sleep Quality Index, MRC: Medical Research Council, INCAT: Inflammatory Neuropathy Cause and Treatment disability scale, I-RODS: Inflammatory Rasch-built Overall Disability Scale, BH: Benjamini-Hochberg, WASO: Wake After Sleep Onset, hr: Heart rate, min: Minimum, max: Maximum

Supplemental Figure 6

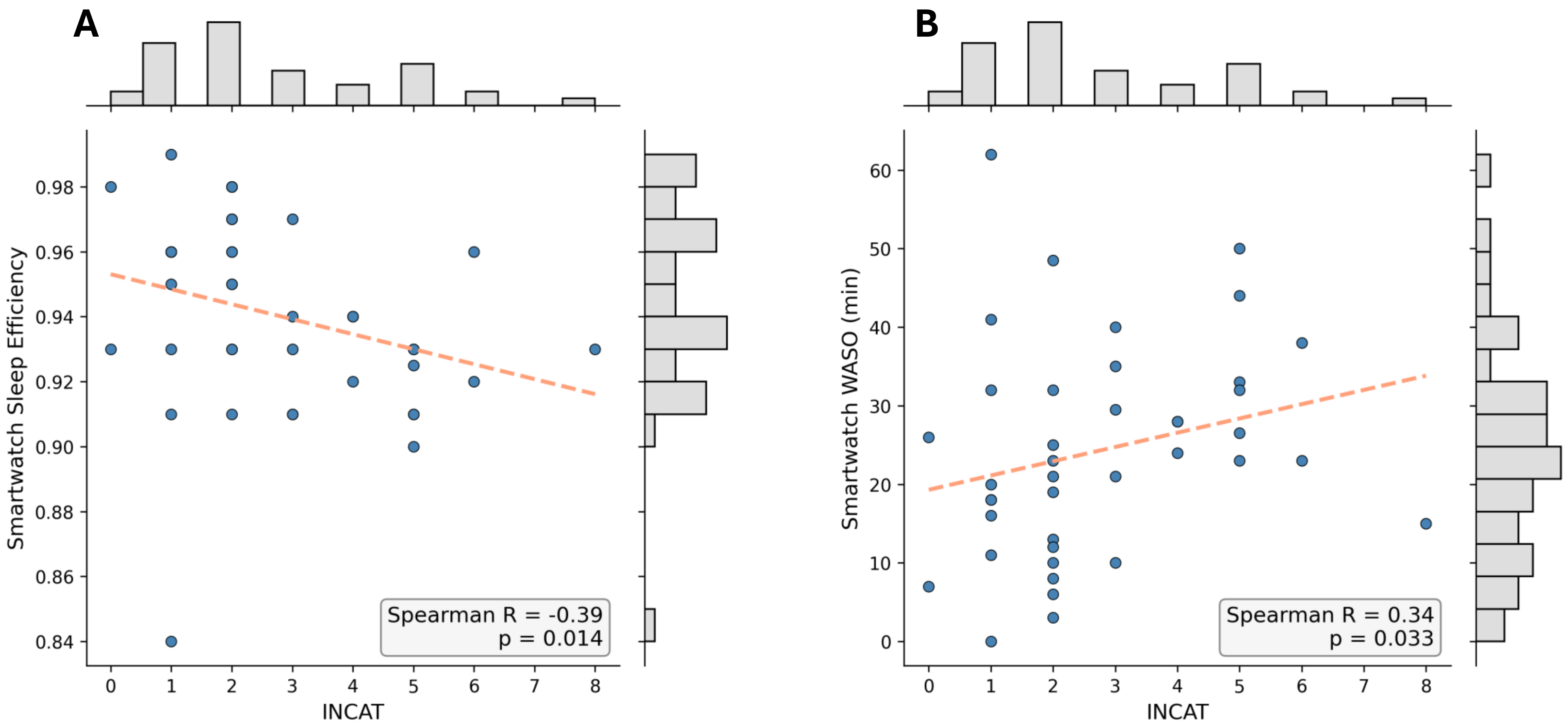

Suppl. Figure 6: Associations between digital sleep parameters and clinical disability score in CIDP patients.

(A, B) Jointplots showing the associations between median INCAT scores and median digital sleep efficiency **(A)** or median WASO **(B)**, including regression lines, marginal histograms, and Spearman correlation coefficients with corresponding p-values. Data points are jittered to enhance visibility. Regression lines are shown in dashed salmon color. Regression lines were only used to visualize correlations, since neither the INCAT nor the sleep efficiency or WASO values were normally distributed.

CIDP: Chronic inflammatory demyelinating polyneuropathy, WASO: Wake After Sleep Onset, INCAT: Inflammatory Neuropathy Cause and Treatment disability score.

Supplemental Figure 7

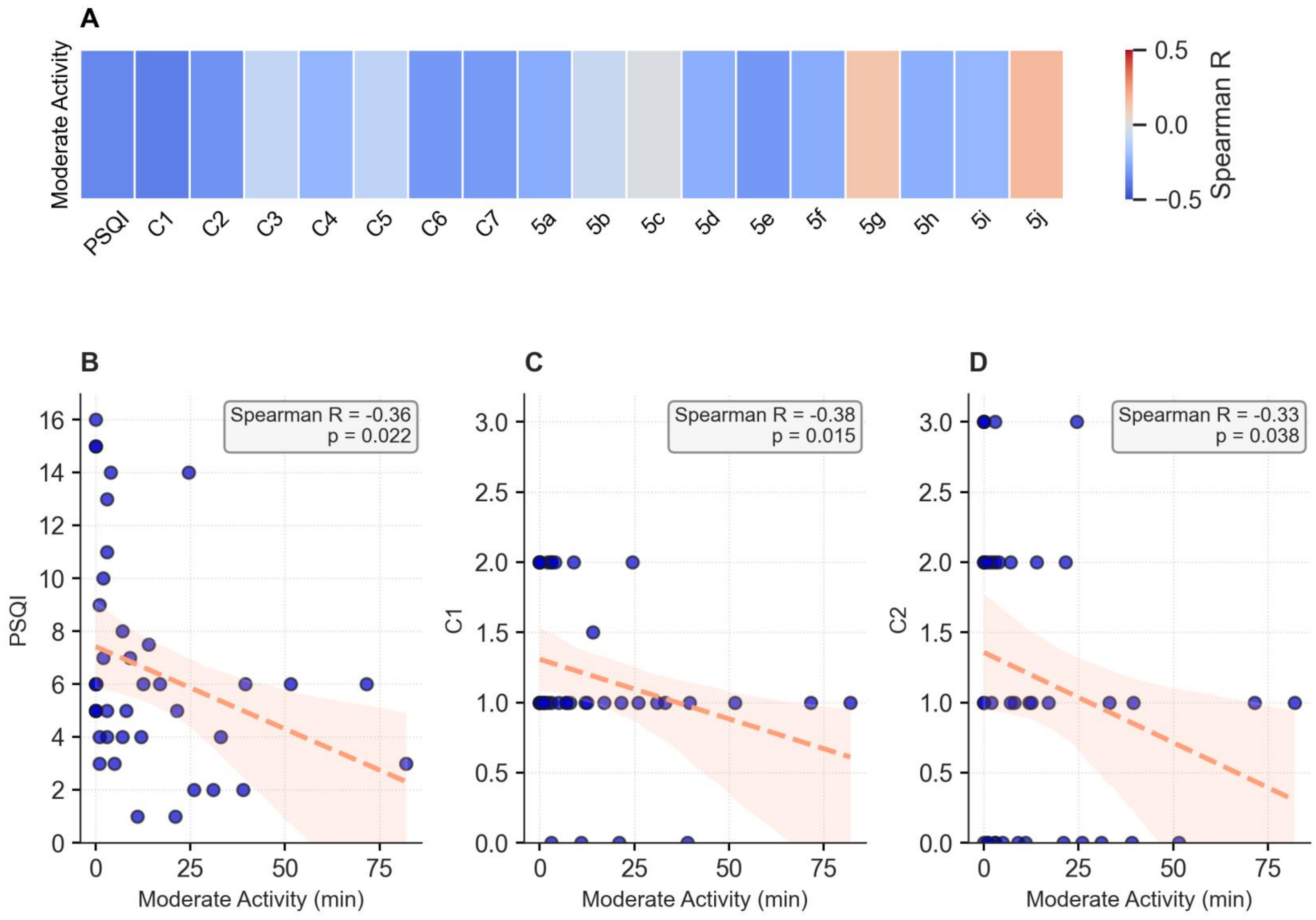

Suppl. Figure 7: Associations between moderate physical activity and PSQI and its subcomponents in CIDP patients.

(A) Heatmap of Spearman correlation coefficients between median moderate activity (minutes per day) and median PSQI scores (PSQI and its subcomponents C1–C7 and in C5: 5a–5j). Correlations were adjusted for multiple comparisons using the Benjamini-Hochberg false discovery rate correction; significant correlations are annotated.

(B–D) Scatterplots with regression lines showing relationships between median moderate activity and median PSQI total score (B), and median PSQI subcomponents C1 (subjective sleep quality, C) and C2 (sleep latency, D). Each plot includes Spearman correlation coefficients and corresponding p-values. Regression lines and 95% confidence intervals are shown in light salmon color. Regression lines were only used to visualize correlations, since neither the moderate activity nor the PSQI values were normally distributed.

CIDP: Chronic inflammatory demyelinating polyneuropathy, PSQI: Pittsburgh Sleep Quality Index, C1: Subjective sleep quality, C2: Sleep latency (including 5a: Falling asleep), C3: Sleep duration, C4: Sleep efficiency, C5: Sleep disturbance, C6: Use of sleep medication (n=5), C7: Daytime dysfunction; C5 subcomponents: 5b: Wake up in the middle of the night or early morning, 5c: Have to get up to use the bathroom, 5d: Cannot breathe comfortably, 5e: Cough or snore loudly, 5f: Feel too cold, 5g: Feel too hot, 5h: Have bad dreams, 5i: Have pain, 5j: Other reason(s)

Supplemental Figure 8

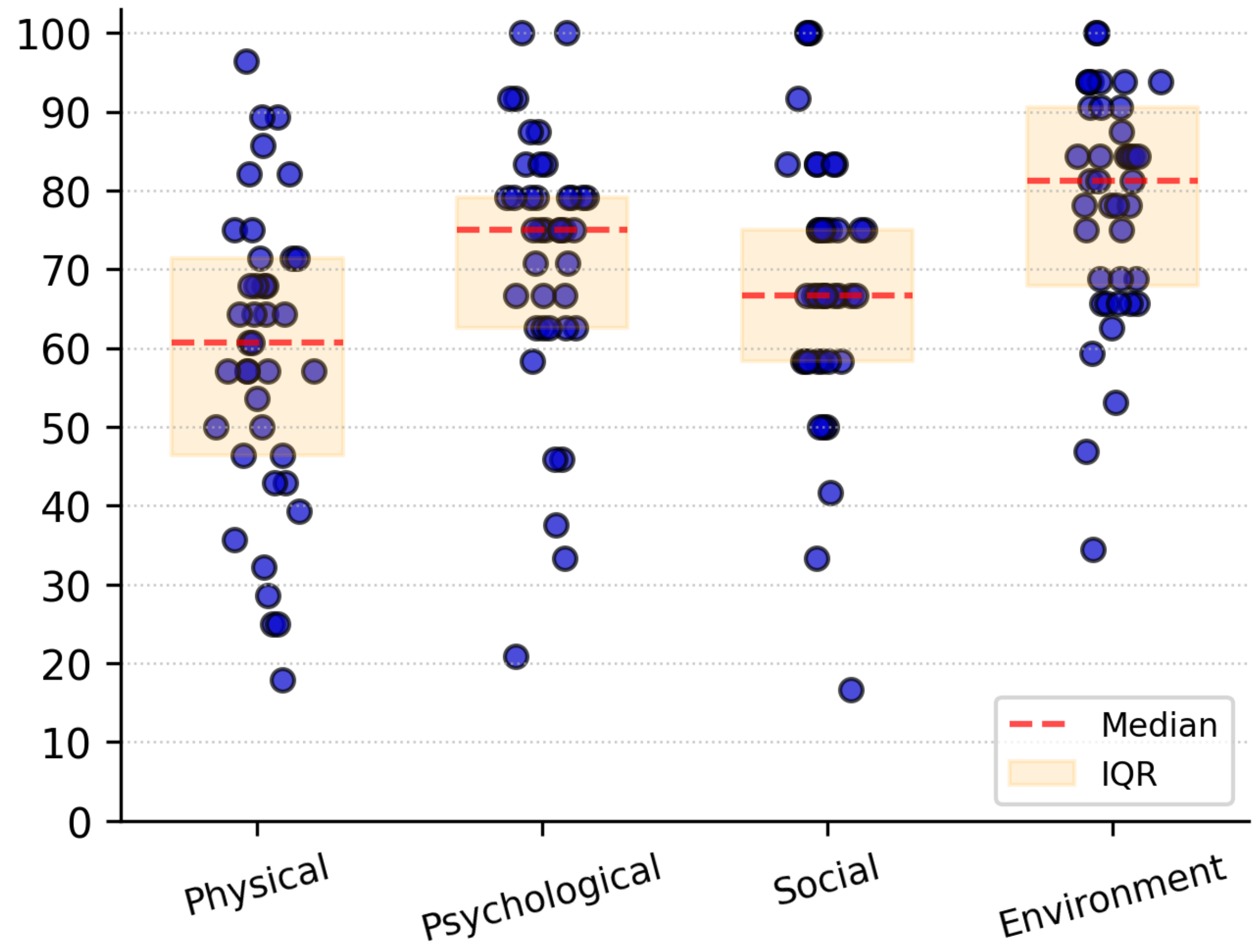

Suppl. Figure 8: Distributions of WHOQOL-BREF domain scores in CIDP patients.

Dot plot showing the distributions of median scores for four WHOQOL-BREF domains: Physical Health, Psychological Health, Social Relationships, and Environment. Each dot represents one patient. Red dashed lines indicate the group median; shaded orange areas represent the interquartile range (IQR). Horizontal jitter was applied to enhance visualization.

CIDP: Chronic inflammatory demyelinating polyneuropathy, WHOQOL-BREF: World Health Organization Quality of Life – abbreviated version.

Supplemental Figure 9

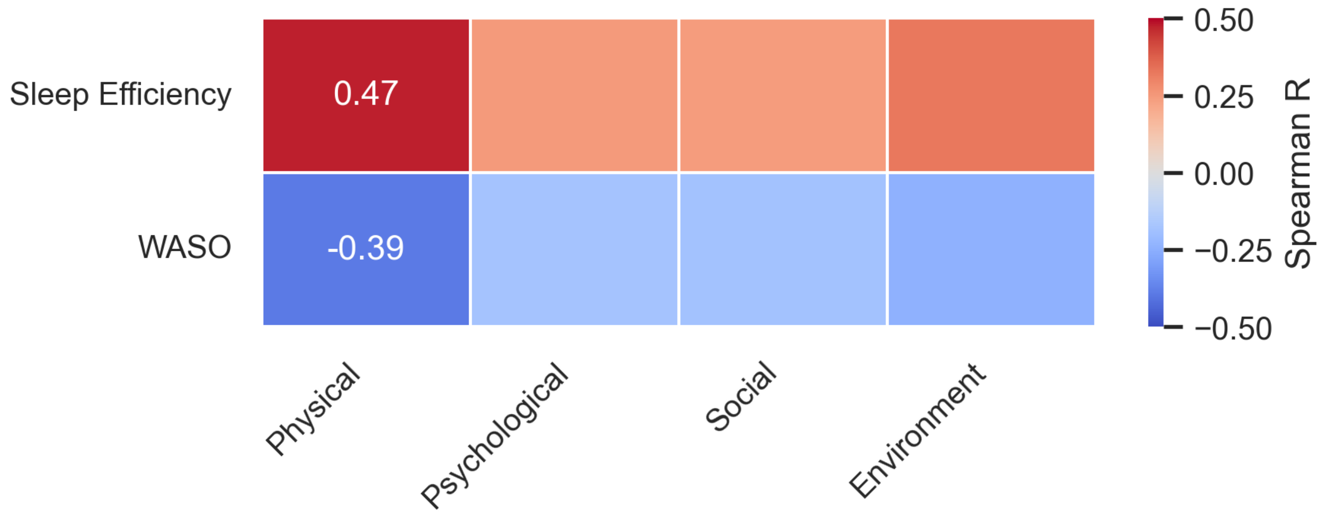

Suppl. Figure 9: Spearman correlations between digital sleep parameters and WHOQOL-BREF domains in CIDP patients after Benjamini-Hochberg correction.

Heatmap showing Spearman correlation coefficients between smartwatch-derived median digital sleep parameters (Sleep Efficiency and WASO) and self-reported WHOQOL-BREF domain scores: Physical Health, Psychological Health, Social Relationships, and Environment. Significant correlations after false discovery rate correction using the Benjamini-Hochberg method are annotated. Color intensity indicates strength and direction of association.

CIDP: Chronic inflammatory demyelinating polyneuropathy, WHOQOL-BREF: World Health Organization Quality of Life – abbreviated version, WASO: Wake After Sleep Onset, BH: Benjamini-Hochberg.
